# The impact of COVID-19 on the dermatologic care of nonmelanoma skin cancers among solid organ transplant recipients

**DOI:** 10.1016/j.jdin.2023.08.005

**Published:** 2023-08-13

**Authors:** Maham Ahmad, Sean R. Christensen, Sara H. Perkins

**Affiliations:** Department of Dermatology, Yale University School of Medicine, New Haven, Connecticut

**Keywords:** COVID-19, nonmelanoma skin cancer, transplant dermatology

*To the Editor:* The COVID-19 pandemic disrupted the dermatologic care of solid organ transplant recipients, who are at increased risk of developing both skin cancers and COVID-19 due to their immunocompromised state.^1^^,^^2^ Given dermatologists’ concern of skin disease progression due to pandemic-related delays in care,^3^ we sought to quantify the impact of the pandemic on the surveillance and treatment of nonmelanoma skin cancers (NMSCs) among transplant recipients.

A retrospective chart review was conducted at Yale University of all solid organ transplant recipients receiving immunosuppressive therapy that were seen by dermatology between January 1, 2019, and December 31, 2021. Data were analyzed using SPSS (version 28.0.0).

In total, 699 patients were included in this study (Table I). During the 3 years, 1217 biopsies were performed, of which 557 were malignant (Table II). There was an increased number of biopsies in 2020 despite a nearly complete lack of in-person visits during early pandemic lockdown from March to May of 2020. Of the NMSCs diagnosed, 8, 16, and 22 of them were found to have high risk features during the years 2019, 2020, and 2021, respectively. There was a significantly higher proportion of high risk malignancies in 2021 (the year after pandemic disruption of care) compared with 2019 (*P* = .02) (Table II). When analyzed by subtype, this significance was found among basal cell carcinomas (BCCs) (*P* = .04), but not squamous cell carcinomas (*P* = .30).

**Table I:** Patient demographics and clinical characteristics

Mean age, y, (SD)	59.14 (13.7)
Gender	
Female	260 (37.2%)
Male	439 (62.8%)
Ethnicity	
Hispanic or Latino	101 (14.4%)
Non-Hispanic	589 (84.3%)
Unknown/other	9 (1.3%)
Race	
American Indian or Alaska Native	11 (1.6%)
Asian	24 (3.4%)
Black or African American	107 (15.3%)
Caucasian or White	449 (64.2%)
Other/unknown	108 (15.5%)
Transplant organ	
Heart	113 (16.2%)
Intestine	1 (0.1%)
Kidney	375 (53.6%)
Liver	166 (23.7%)
Lung	9 (1.3%)
Multiple organs	35 (5.0%)
Immunosuppressive treatment	
Azathioprine	46 (6.6%)
Belatacept	75 (10.7%)
Cyclosporine	106 (15.2%)
Everolimus	5 (0.7%)
Mycophenolate mofetil/mycophenolic acid (Myfortic)	414 (59.2%)
Prednisone	405 (57.9%)
Sirolimus/rapamycin	37 (5.3%)
Tacrolimus	510 (73.0%)

**Table II:** Biopsies and cutaneous malignancies by year

Patient demographics and clinical characteristics	2019	2020	2021	Total
Biopsies	390	434	393	1217
Malignancies∗	170	197	190	557
SCC	60	58	59	177
SCCIS	62	75	76	213
BCC	44	59	52	155
Other	4	5	3	12
Malignancies with high risk features†	8 (4.7%)	16 (8.1%)	22 (11.6%)	46 (8.3%)
SCC	7	12	14	33
BCC	1	4	8	13
Cancers treated with Mohs micrographic surgery‡	62 (36.5%)	67 (34.0%)	82 (43.2%)	211 (37.9%)
Cancers treated at time of biopsy‡	18 (10.6%)	43 (21.8%)	36 (18.9%)	97 (17.4%)
SCC	7	9	6	23
SCCIS	5	16	12	33
BCC	6	18	18	41
H area	1	2	2	5
M area	9	3	2	14
L area	8	38	32	78

*BCC*, Basal cell carcinoma; *SCC*, squamous cell carcinoma; *SCCIS*, squamous cell carcinoma *in situ*.

∗ χ^2^ analysis: proportion of biopsies found to be malignant.

† χ^2^ analysis: proportion of skin cancers found to have high risk features. High risk features were defined as: aggressive features on histology (SCC: infiltrating, acantholytic, and poorly differentiated; BCC: infiltrating and micronodular), perineural invasion, depth of invasion beyond subcutis, and/or metastasis.

‡ χ^2^ analysis: proportion of skin cancers that received this treatment.

To determine trends that occurred after the initial COVID-19 lockdown, the time periods of March to December of each year were analyzed. There was no significant difference in proportion of biopsies found to be malignant (*P* = .66) or proportion treated with Mohs micrographic surgery (*P* = .31). However, there was a significant increase in the proportion of NMSCs treated at the time of biopsy in 2020 (*P* < .001); further analysis found this increase among squamous cell carcinoma *in situ* (*P* = .01) and BCC (*P* = .02) subtypes, and skin cancers in low risk “L” anatomic regions (*P* < .001) as defined by Mohs micrographic surgery appropriate use criteria.^4^

When comparing NMSCs biopsied between January and March of 2019 versus 2020, there was a significant delay to Mohs micrographic surgery in 2020 (126.0 vs 53.5 days, *P* = .03), but there was no significant difference in postoperative defect size (*P* = .34) or number of stages (*P* = .39) (Supplementary Table I, available via Mendeley at https://data.mendeley.com/datasets/kjtdt5khtg/1).

A recent study found no difference in rates of NMSC during the pandemic but did not comment on high risk features.^5^ In our study of transplant recipients, we found a significant increase in NMSCs with high risk features in 2021; this trend was observed for both BCC and squamous cell carcinoma but only reached statistical significance for BCC. It is not clear if the increased incidence of high risk cancers was due to delayed treatment, increased detection, or other unknown factors. Of note, 2 skin cancers that were treated with electrodessication at the time of biopsy in March 2020 subsequently recurred, raising the concern that lack of definitive excision increased risk of recurrence.

Limitations include the single institution and retrospective nature of this study. Larger, multiinstitutional studies could further assess the effects of the COVID-19 pandemic on dermatologic care of transplant recipients.

## Conflicts of interest

None disclosed.
